# Quetiapine, an Atypical Antipsychotic, Is Protective against Autoimmune-Mediated Demyelination by Inhibiting Effector T Cell Proliferation

**DOI:** 10.1371/journal.pone.0042746

**Published:** 2012-08-13

**Authors:** Feng Mei, Sheng Guo, Yangtao He, Linyun Wang, Hongkai Wang, Jianqin Niu, Jiming Kong, Xinmin Li, Yuzhang Wu, Lan Xiao

**Affiliations:** 1 Department of Histology and Embryology, Chongqing Key Laboratory of Neurobiology, Third Military Medical University, Chongqing, China; 2 Department of Immunology, Third Military Medical University, Chongqing, China; 3 Department of Human Anatomy and Cell Science, University of Manitoba, Winnipeg, Canada; 4 Department of Psychiatry, University of Manitoba, Winnipeg, Canada; Emory University, United States of America

## Abstract

Quetiapine (Que), a commonly used atypical antipsychotic drug (APD), can prevent myelin from breakdown without immune attack. Multiple sclerosisis (MS), an autoimmune reactive inflammation demyelinating disease, is triggered by activated myelin-specific T lymphocytes (T cells). In this study, we investigated the potential efficacy of Que as an immune-modulating therapeutic agent for experimental autoimmune encephalomyelitis (EAE), a mouse model for MS. Que treatment was initiated on the onset of MOG_35–55_ peptide induced EAE mice and the efficacy of Que on modulating the immune response was determined by Flow Cytometry through analyzing CD4^+^/CD8^+^ populations and the proliferation of effector T cells (CD4^+^CD25^−^) in peripheral immune organs. Our results show that Que dramatically attenuates the severity of EAE symptoms. Que treatment decreases the extent of CD4^+^/CD8^+^ T cell infiltration into the spinal cord and suppresses local glial activation, thereby diminishing the loss of mature oligodendrocytes and myelin breakdown in the spinal cord of EAE mice. Our results further demonstrate that Que treatment decreases the CD4^+^/CD8^+^ T cell populations in lymph nodes and spleens of EAE mice and inhibits either MOG_35–55_ or anti-CD3 induced proliferation as well as IL-2 production of effector T cells (CD4^+^CD25^−^) isolated from EAE mice spleen. Together, these findings suggest that Que displays an immune-modulating role during the course of EAE, and thus may be a promising candidate for treatment of MS.

## Introduction

Disseminated demyelination in the central nervous system (CNS) mediated by autoimmune reactive inflammation is the primary pathological hallmark in multiple sclerosis (MS) and in various animal models, including experimental autoimmune encephalomyelitis (EAE), which results in axonal injury, synaptic dysfunctions and neurological impairments [1], [2]. The activated myelin-specific CD4^+^ lymphocytes (T cells) that infiltrated into CNS nerve tissue have been considered as the initiator or early effector cells in the development of both EAE and MS [3], [4].

For therapy of MS or EAE, a series of strategies including immunoregulation, anti-inflammation and enhancement in neuroprotection and/or neuroregeneration mediated by small molecules or mesenchymal stem cells have been applied to re-balance/control the immune response and protect myelin from breakdown [5]–[9]. Currently, interferon β (IFNβ) and glatiramer acetate (GA) are commonly used to treat MS [10], [11]. These drugs act mainly to rebalance the immune response and are capable of slowing down disease progress and ameliorating the frequency of recurrence of MS, but the efficacy is still limited and the long-term outcome is not satisfactory, most likely due to the inefficiency in myelin repair and neuronal degeneration [1]. Therefore, to rescue myelin from breakdown and to promote remyelination should be incorporated into the approach and treatment of demyelinating diseases including MS.

Quetiapine (Que) is a commonly used atypical antipsychotic drug (APD) that has superior therapeutic effects on negative and cognitive symptoms in patients with schizophrenia and other neurological disorders like depression. Previous *in vivo* and *in vitro* studies have demonstrated that Que exerts protective effects on neurons [12]. Recently, oligodendrocyte dysfunction or demyelination has been implicated in the pathophysiology of schizophrenia, bipolar disorder and major depression [13]. In trying to reveal the cellular mechanisms underlying the therapeutic actions of Que, recent studies indicate that Que can effectively prevent myelin breakdown in the cerebral cortex and the concomitant spatial working memory impairment in cuprizone induced demyelination mouse model without immune attacks [14]–[16]. Furthermore, Que can inhibit the activation of microglia in the brain of these mice [16], [17]. These observations suggest that Que may address two key aspects involved in the pathophysiological process of MS, namely prevention of demyelination and modulation of the local glial activation, which suggests broader potentials for Que in demyelination diseases. However, it is still unknown whether Que displays an immune-modulating role and may be a promising candidate for treatment of MS.

To investigate the potential efficacy of Que as a therapeutic agent, we utilized the MOG induced EAE mouse model to mimic MS. We demonstrate that Que dramatically attenuates the severity of EAE symptoms, diminishes demyelination and the infiltration of CD4^+^/CD8^+^ T cells, as well as activation of local microglia in the spinal cord. Additionally, our results indicate that Que exerts immunomodulatory capacities to attenuate MOG_35–55_-specific immune response, and to inhibit effector T cell proliferation and thus reduce peripheral CD4^+^/CD8^+^ T population as administrated to EAE mice. Overall, our findings demonstrate the utility of Que as a potential therapeutic agent for demyelinating diseases such as MS.

**Figure 1:**
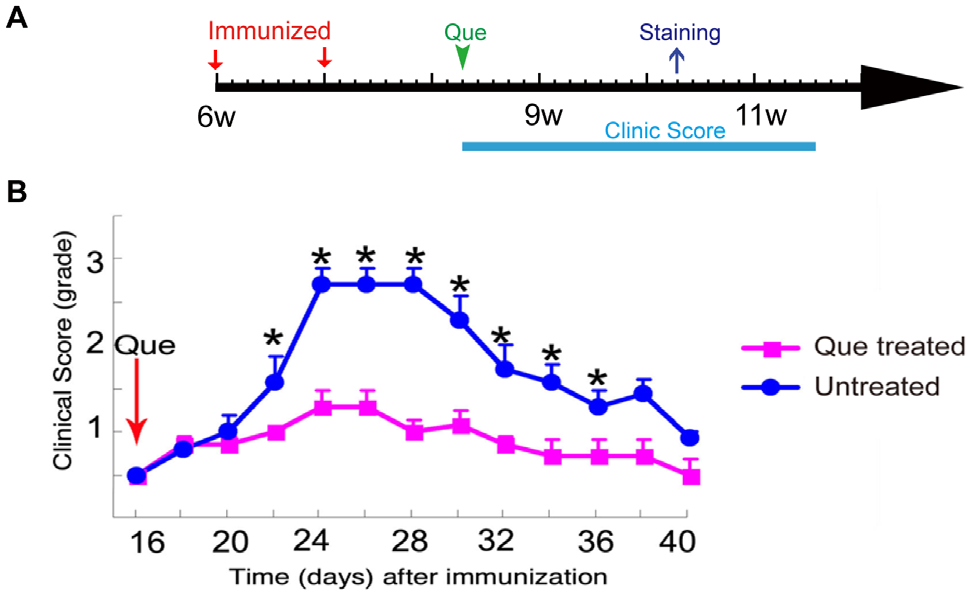
Post-treatment of Que rescues EAE mice from deterioration. **A**: Schematic diagram displaying the time course of immunization and Que post-treatment. **B:** Que post-treatment was initiated on day 16 post-immunization when mice attained a clinical score of 0.5 (arrow). Untreated mice (N = 9) continued to deteriorate with increasing clinical scores that reached values of approximately 3. Mice treated with Que (N = 9) with an initial score of 0.5 reached a value just greater than1, and were stabilized and maintained at that value. Values shown are means ± SEM (*, *p<0.05*).

## Materials and Methods

### EAE Mice Model and Treatment Protocols

C57BL/6 mice were obtained from the Animal Center of Third Military Medical University. All experiments were performed in accordance with Health Guide for the Care and Use of Laboratory Animals, with the approval of Third Military Medical University Committee on Animal Care (permission NO: SCXK-JUN-2007-015). Female C57BL/6 mice (N = 43, 8 weeks old) were immunized subcutaneously with 200 µg of MOG_35–55_ peptide (Invitrogen, Carlsbad, CA) emulsified in complete Freund adjuvant (CFA, Sigma Aldrich, Saint Louris, MO) on Day 0 and Day 7, and received 300 ng pertussis toxin (PT, List Biological Laboratories, Campbell, CA) in 0.1 ml PBS intraperitoneal at the time of immunization and 48 hours later. The control (N = 10) mice were immunized with bovine serum albumin (BSA) at the same dosage (200 µg) followed by PT and the unimmunized mice (N = 9) were given only PBS and PT. Onset and clinical scores of EAE symptoms were evaluated daily using a neurological score as follows: 0, no clinical signs; 0.5, partially limp tail; 1, paralyzed tail; 2, loss in coordinated movement; hind limb paresis; 2.5, one hind limb paralyzed; 3, both hind limbs paralyzed; 3.5, hind limbs paralyzed with weakness in forelimbs; 4, forelimbs paralyzed; 5, moribund as previously described [18].

### Quetiapine Treatment Protocols

Quetiapine (AstraZeneca, Wilmington, DE) (dissolved in distilled water) was orally administrated to the mice (10 mg/kg/day) [14]. Que was administrated orally in EAE groups (Que treated) on Day 16 after immunization and maintained for 24 days to test clinical symptoms (40 days after immunization) and histology changes were detected on 30 days after immunization, a time at which apparent histopathology changes can be detected, while those in the untreated groups were given only distilled water. To test immunoresponse in periphery immune organs, Que was orally administrated 1 week before immunization, and mice were sacrificed on Day 10 after immunization, a time at which very efficient MOG_35–55_-specific responses can be detected.

**Figure 2:**
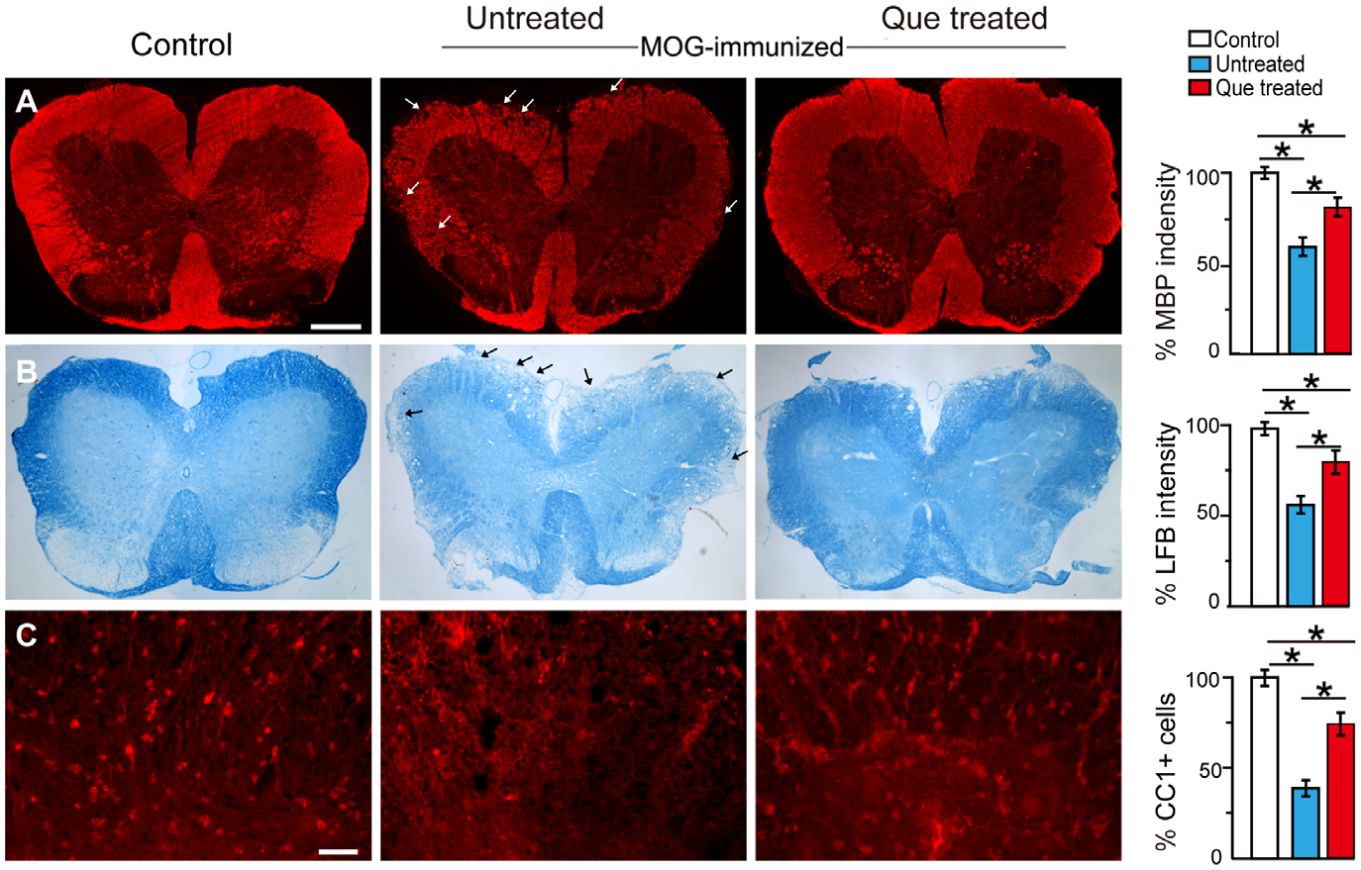
Que protectes the spinal cord from demyelination and loss of oligodendrocytes. **A.** MBP immunofluorescent staining displays an obvious decrease of myelin (arrows) in the white matter of the spinal cord in the untreated EAE controls (N = 5). Post-treatment with Que (N = 5) protects myelin from breakdown and displays a similar fluorescence intensity to the unimmunized controls (N = 5). **B.** Luxol Fast Blue (LFB) staining indicates a similar pattern after Que treatment (arrows). **C.** Que prevents the loss of CC1^+^ oligodendrocytes as compared with the untreated controls. Quantification of the observations is provided in the bar graphs. (*, *p<*0.05). Scale bar A = B = 0.5 mm, C = 0.2mm.

### Immunocytochemical Staining and Quantification

On Day 30 after immunization, mice were deeply anesthetized with 1% pentobarbital and transcardially perfused with 4% paraformaldehyde in PBS. Spinal cords were dehydrated in 30% sucrose and crossly cut (20 µm) using a cryostate microtome (MS 1900, Leica). Sections were blocked with 10% BSA and incubated with rat anti-CD4, CD8, CD68, CD_11b_ antibodies, or goat anti-MBP, rabbit anti-NG2, mouse anti-APC, and mouse anti-GFAP antibodies (Table S1) overnight at 4°C followed by using an Alex Fluor 488, 568 or Cy5-conjugated secondary antibody relatively. The results were examined under a fluorescence microscope (90 i, Nikon,) or a laser confocal scanning microscope (PV100, Olympus) with the excitation wavelengths proper for Alex Fluor 488 (488 nm), Alex Fluor 568 (568 nm), Cy5 (628 nm) or DAPI (380 nm). For stereological quantification, serial sections of spinal cord from L1–L6 were collected (about 200 sections) and twenty sections were sampled from each animal in a systematic and random manner. In practice, every first and the tenth sections were sampled from the 200 sections systematically. After immunofluorescence staining, digital images of CD4+, CD8+, CC1+, NG2+, GFAP+, CD8+, CD11b+, CD68+ and MBP staining were acquired with a digital camera (Nikon, Japan) mounted on a 90 i fluorescence microscope (Nikon, Japan). The cell numbers of CD4, CD8, CC1 and NG2 positive cells and the mean intensity of CD11b+, GFAP+, CD68+, MBP+ and Fast Blue staining were quantified with Image-Pro Plus 5.0 (Media Cybernetics, Silver Spring, MD, USA).

### Flow Cytometry Analysis

Mice were anesthetized, spleen and draining lymph nodes removed, and single cell suspension was prepared. Cells isolated from lymph nodes and spleens were adjusted at 1×10^6^ cells/vial and stained with Percp cy5.5-anti-CD4(clone RM4-5), PE-anti-CD25(clone PC61.5) or Percp cy5.5-anti-CD8 (clone 53-6.7,) for 30 min at 4°C, then washed with PBS containing 1% FCS. All antibodies were obtained from eBioscience. In some instances, isotype-matched IgG were used as negative controls. In each test, 1,000,000 cells were collected by a Canto II flow cytomerer using Cell Quest Diva software (BD Biosciences) and analyzed by FlowJo software (TriStar).

**Figure 3:**
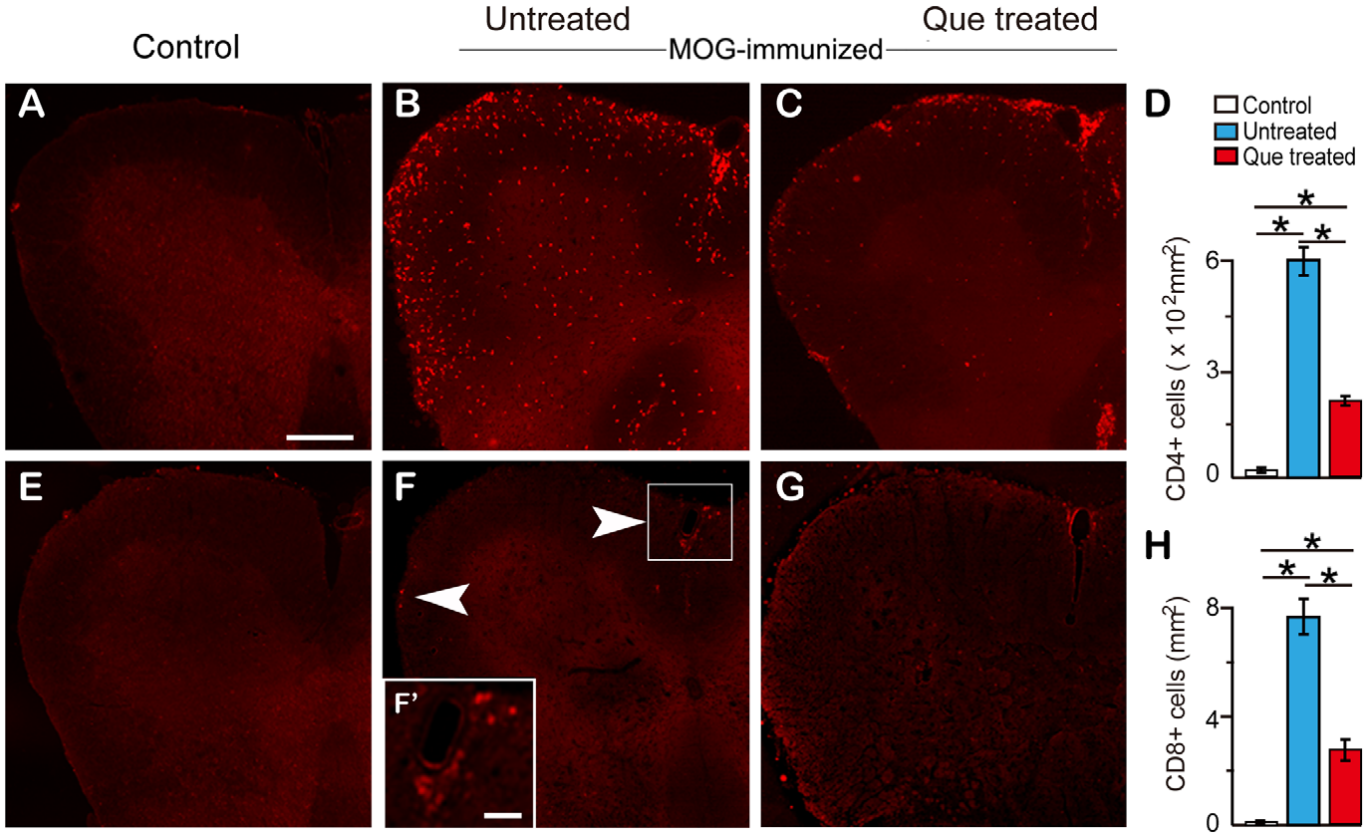
Que decreases the number of infiltrating T cells in spinal cord. Panels display the number of infiltrating CD4/8 positive T cells in the EAE spinal cord with/without Que treatment. **A–C**: CD4^+^ cells were not observed in the spinal cord prior to immunization (**A**). An extensive infiltration of CD4^+^ cells was detected throughout the spinal cord and enriched in the white matter of the EAE mice (**B**). Que treatment significantly decreases the amount of infiltrating CD4^+^ T cells (**C**) (N = 5, *, *P<*0.05). **E–G**: CD8^+^ cells display a similar decrease after Que treatment (N = 5, *, *p<*0.05), but the overall extent of CD8^+^ cells detected was much less than that of CD4^+^ cells. Quantification of the staining is depicted in the bar graphs (**D and H**). Scale bar **A–G** = 0.5 mm, **F’** = 50 µm.

### CFSE Proliferation Assay

Proliferation of T cells was measured by dilution of the dye CFSE. To detect MOG_35–55_-specific proliferation of T cells, single cell suspensions from spleen were obtained at 10 days after MOG_35–55_-immunization. Cells were incubated with CFSE (2 µM) (eBioscience) for 10 min at 37°C in the dark, then washed with cold complete media 3 times. Cells labeled with CFSE were cultured in 96-well plates (2×10^5^ cells/well) and stimulated with indicated concentration of MOG_35–55_ peptide in triplicate at 0, 1, 10 or 100 µg/ml. In additional experiments, CD4^+^CD25^−^ T cells in spleens of naïve C57BL/6 mice (N = 10) were fractionated using magnetic bead chromatography (Miltenyi). Purity of the samples was routinely tested after sorting and was >95%. Cells were the labeled with CFSE and cultured in 96-well plates (2×10^5^ cells/well) coated with anti-CD3 antibody (1 µg/ml) by adding Que (1 µM) or not for 72 h. The results were analyzed using FACS (Canto II, BD).

### RNA Preparation and Real-time PCR

CD4^+^CD25^−^ T cells were cultured in 96-well plate coated with anti-CD3(1 µg/ml), incubating with/wihout Que(1 µM) for different time (0, 12, 24 or 48 h), total RNA was extracted with Trizol Reagent (Invitrogen) following the manufacture’s instructions after indicated time. Reverse transcription of RNA was performed using an RNA PCR Kit (AMV) (Takara). The cDNA was analyzed by real-time PCR with the Rotor Gene6000 (Corbett Research, Australia) according to the protocol provided by the manufacturer and 2^−ΔΔCt^ method. Briefly, PCRs were performed using SYBR premix Ex Taq (Takara) in a final volume of 20 µl. The thermal conditions were 95°C for 10 seconds followed by 40 cycles of 95°C for 5 seconds, 60°C for 15 seconds and 72°C for 15 seconds. The primers were IL-2 sense 5′-CATTGACACTTGTGCTCCTTG-3′, antisense 5′-GGTTCCTGTAATTCTCCATCCTG-3′, mouse actin-β sense 5′-CGTGCGTGACATTAAGGAGAAG-3′, antisense 5′-GGAAGGAAGGCTGGAAGAGTG-3′.

### Detection of IL-2 by ELISA

The supernatants were obtained and examined by ELISA (eBioscience). In brief, serially diluted supernatants samples and internal standards samples (recombinant murine IL-2) were incubated with immobilized antibody. Antibody was detected with HRP-labeled rabbit anti-mouse IgG1 and IgG2a (Zymed Laboratories), which were then detected with the substrate o-phenylenediamine. The relative concentration of antibody was determined from a standard curve of known concentrations of unlabeled murine IL-2 antibody (Southern Biotechnology Associates).

### Statistical Analysis

Data were expressed as means ± SEM. Statistical analysis between experimental groups was evaluated using analysis of variance (ANOVA). A two-tailed paired Student’s t test was used for comparing individual treatment groups. A probability of *p*<0.05 was taken as a statistically significant difference.

**Figure 4:**
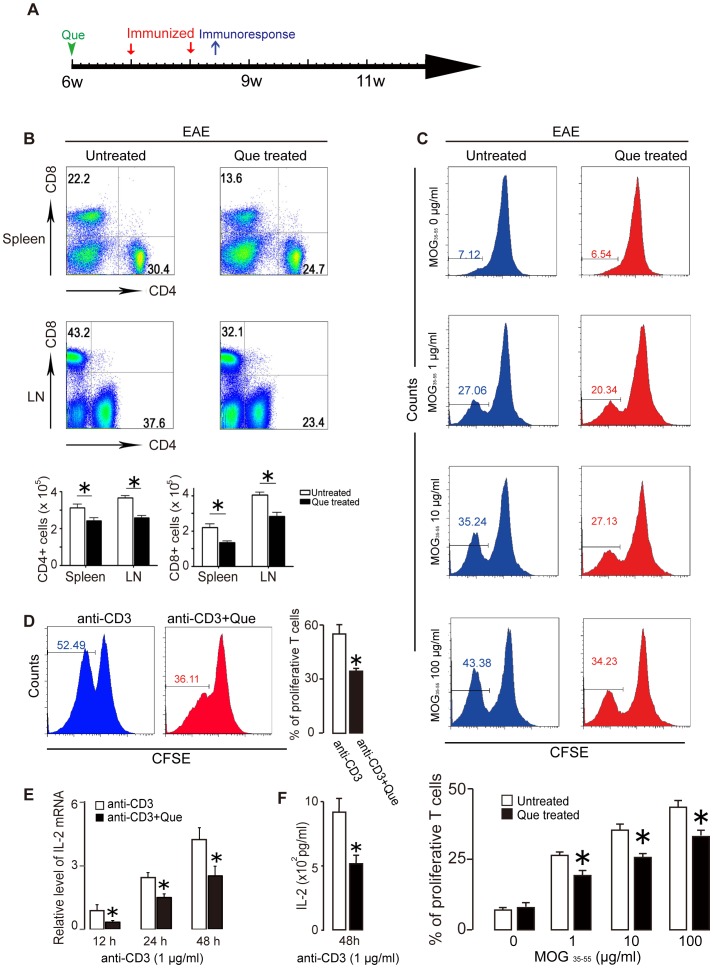
Que treatment decreases the number of T cells in the spleen and lymph nodes and inhibites proliferation of effector T cells. **A.** Schematic diagram displaying the time course of immunization and Que pre-treatment. **B.** CD4^+^/CD8^+^ cells in the spleen and lymph nodes were quantified by FACs after Que treatment. The scatter plot (lower panel) shows the number of CD4^+^/CD8^+^ cells per million cells. In both cases, the number of cells was decreased upon Que treatment. **C.** Mononuclear cells isolated from the spleens of mice were immunized with MOG_35–55_ peptide with or without Que treatment, and were stimulated with 0, 1, 10 or 100 µg/ml of MOG35–55 peptide. The proliferation was significantly suppressed assayed by CFSE. **D.** Effector T cells (CD4^+^CD25^−^) isolated from spleen of naïve mice are labeled with CFSE. After stimulation with anti-CD3, dilution of CFSE shows that CD4^+^CD25^−^ populations proliferated much less vigorously after Que treatment than vehicle. **E–F.** IL-2 expression in effector T cells is decreased after Que-treatment assayed by either real-time PCR (E) or ELISA (F). Data represent mean ± SEM of five independent experiments (N = 5, *, p<0.05).

## Results

### Que Administration Attenuates Clinical Signs and Protects Myelin from Breakdown in EAE

Que treatment was initiated on day 16 post-immunization when mice attained a clinical score of 0.5, and mice were monitored for a total of 40 days (Fig. 1A). The untreated EAE mice continued to deteriorate with increased clinical scores that reached a value of approximately 3, whereas mice treated with Que reached a value of 1, then stabilized at that level and did not display further deterioration (Fig. 1B). While unimmunized group and control (immunized with BSA) mice displayed normal behavior (data not shown). Therefore, Que treatment clearly attenuates the clinical scores in EAE. Additionally, pre-treatment with Que from 1 week before immunization resulted in a significant delay in the onset of EAE and diminishes the severity of symptoms (Figure S1).

As demyelination is one of the major histopathologic hallmarks in EAE and MS, histological observation indicate that the spinal cords from the immunized group without Que treatment were weakly stained with MBP and LFB, especially in the white matter tracts (arrows, Fig. 2A, B), while a more intense expression of MBP and LFB staining were observed in the Que treatment group but less intense than unimmunized group or control (Fig. 2A, B) on 30 days after immunization. Moreover, CC1^+^ oligodendrocytes (OLs) were dramatically decreased in the EAE mice with or without Que treatment as compared to unimmunized animals, while these cells were more conserved in the Que treatment group (Fig. 2C). Ki67 was used to detect the proliferative oligodendrocyte precursors (OPCs) (NG2^+^) and a number of Ki67^+^/NG2^+^ cells were seen distributed throughout the white matter in the EAE model (Figure S2). Such cells were also detected after Que treatment, however likely, to a lesser extent (Figure S2), while such proliferative OPCs were rare in normal controls. Together, these results indicate that Que treatment protects the spinal cord from demyelination and loss of OLs in EAE mouse models and OPC proliferation is a prominent response to demyelination.

### Que Reduces Infiltration of T Cells in the EAE Spinal Cord

Since myelin-specific effector T cells migrate into CNS and initiate demyelination [4], dose the treatment of Que affect the accumulation of T cells in the spinal cord of EAE model? The immunohistochemical results indicate that both CD8^+^ and CD4^+^ T cells were barely detected in unimmunized mice and control (Fig. 3A, E), while a large number of CD4^+^ T cells were found to accumulate in the spinal cord of the EAE model (Fig. 3B), especially distributed throughout the white matter. These cells were significantly decreased in the spinal cord of the Que-treated group (Fig. 3C–D). The CD8^+^ T cells were also observed infiltrated into spinal cord (Fig. 3F) and were reduced after Que treatment (Fig. 3F–H) with the exception that the number of CD8+ T cells was much fewer than that of CD4^+^ cells.

### Que Attenuates MOG_35–55_-specific Immune Response and Inhibits Effector T Cell Proliferation

The myelin-specific effector T cells are generated in periphery lymph organs. To confirm the reduction of CD4^+^ and CD8^+^ T cells found in the spinal cord and to reveal their source, we detected the CD4^+^/CD8^+^ T cell numbers from spleen and lymph nodes using FACS 10 days after immunization with Que pre-treatment for 1 week (Fig. 4A) and found that Que treatment resulted in reduced numbers of CD4^+^ and CD8^+^ T cells as compared with untreated EAE mice (Fig. 4B). To test whether Que attenuated MOG_35–55_-specific T cells generation, we measured the *in vitro* stimulated proliferation of T cells isolated from spleens 10 days after MOG_35–55_ immunization, with or without Que pre-treatment. The results show that MOG_35–55_-stimatuating proliferation of T cells from primed mice spleen were significantly decreased in Que treated mice (Fig. 4C).

To further examine whether the reduction of T cells comprise a non-specific proliferation inhibitive effect by Que, we isolated CD4^+^CD25^−^ T cells (effector T cells) from spleen of naïve C57BL/6 mice and stimulated the cells with anti-CD3 Ab with/without Que treatment, dilution of CFSE results exhibit that CD4^+^CD25^−^ T cells from Que treatment proliferated much less vigorously than that from untreated EAE mice (Fig. 4D). Additionally, Que decrease the expression IL-2, a potent T cell growth factor, assayed by either real-time PCR (Fig. 4E) or ELISA (Fig. 4F). Together, these results indicate Que can either attenuate MOG_35–55_-specific immune response or inhibit effector T cell proliferation.

### Que Diminishes Accumulation of CD4^+^ T Cells that Reduces OLs Loss and Demyelination in EAE Spinal Cords

**Figure 5:**
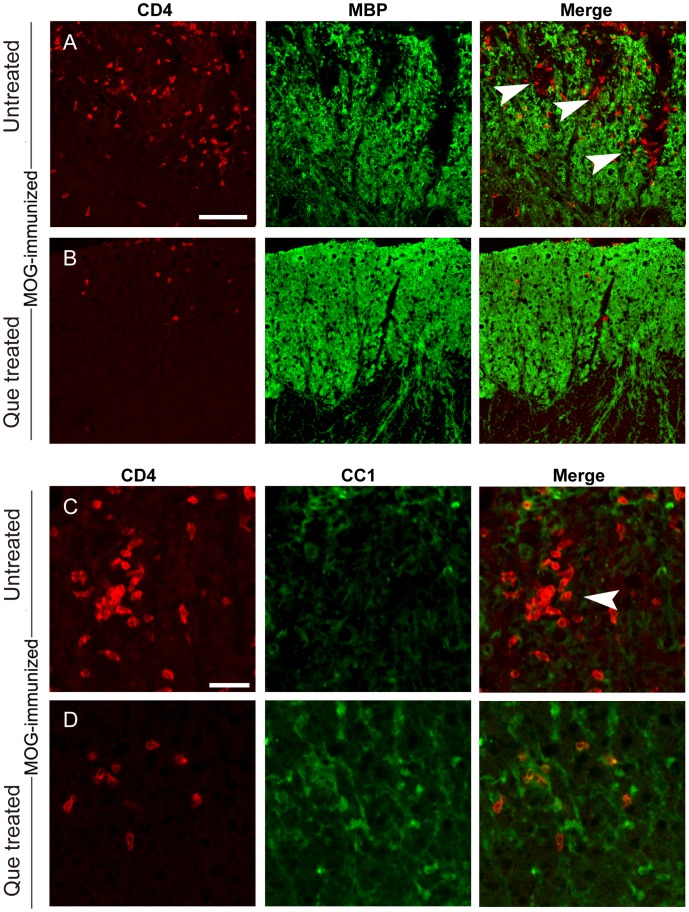
Que decreases the number of infiltrating T cells and protects mice from demyelination and OLs loss. Micrographs display the number of infiltrating CD4^+^ cells (red, **A–D**), MBP expression (Green, **A,B**) and oligodendrocytes (CC1^+^, Green, **C,D**) in the spinal cord of the EAE model with/without Que treatment. **A–D**: Clusters of infiltrating CD4^+^ cells are observed in the spinal cord without Que treatment (**A, C**) and, surrounding these areas, demyelination is present (arrowheads, **A**) and is devoid of oligodendrocytes (CC1^+^, arrowheads, **C**); Que treatment decreases CD4^+^ cell infiltration (**B, D**) and more abundant myelin segements (MBP^+^, **B**) and oligodendrocytes (CC1^+^, **D**) are observed surrounding individual CD4^+^ cells. Scale bar **A–B** = 0.1 mm; **C–D** = 80 µm.

As the infiltrated T cells initiate OLs damage and local immunoresponse, dose reduced CD4^+^ T cells rescue demyelination and protect OLs from loss? The double labeling experiments with CC1/MBP and CD4 showed CD4^+^ cells were abundantly distributed in the white matter upon EAE induction (Fig. 5A,C, arrowhead), and were specifically localized to demyelination lesions (Fig. 5A), while CC1^+^ OLs were absent from these areas (Fig. 5C). These observations suggest that the accumulation of CD4^+^ cells could lead to loss of OLs and demyelination. Treatment of Que, however, decreased the number of infiltrated CD4^+^ cells, with a subsequent increase in the number of CC1^+^ OLs (Fig. 5D), observed adjacent to a single appearing CD4^+^ T cell, accompanied with normal myelin staining (Fig. 5B).

### Que Inhibits Activation of Microglia and Astrocytes in EAE Spinal Cord

**Figure 6:**
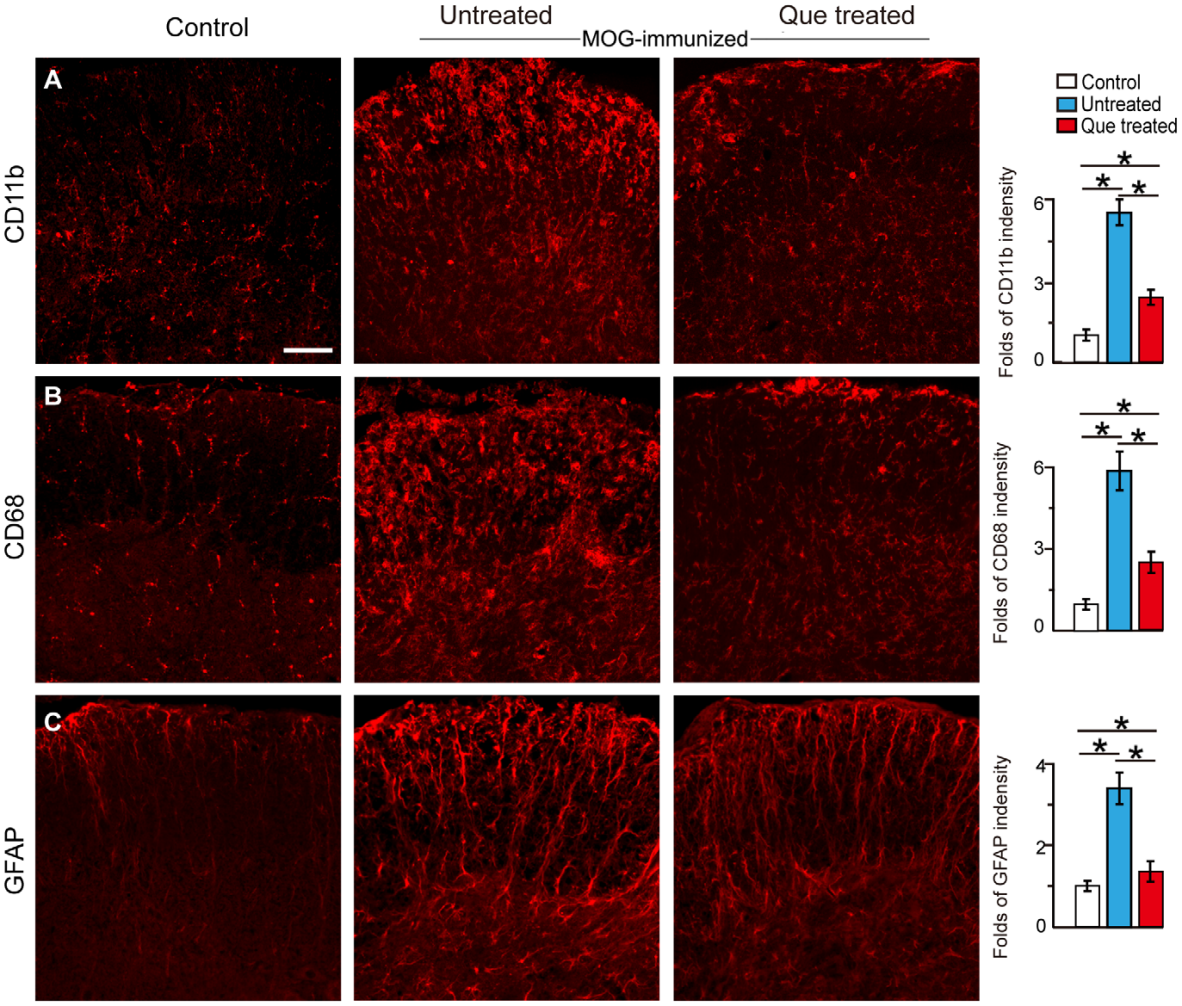
Que inhibits the activation of microglia/macrophages and astrocytes in spinal cord. Immunofluorescent staining with anti-CD68 and CD11b antibodies to demonstrate the number of microglia/macrophages in the EAE model with/without Que treatment, A. Numerous CD11b^+^ microglia/macrophages were observed in the EAE model which were greatly decreased after Que treatment. B. CD68 staining displayed a similar staining pattern after Que treatment. C. GFAP immunofluorescent staining displayed an increase in reactive astrocytes in the spinal cord, which was greatly decreased after Que treatment (N = 5, *, *p<*0.05). Quantification of the immunostaining is present in the bar graphs. Scale bar A–C = 0.2 mm.

As local immunoresponse is initially triggered by infiltrated T cells and contributed to the neuronal damage and demyelination, we observed CD68^+^ and CD11b^+^ cells which represent activated microglia/macrophages, were extensively populated throughout the spinal cord in the EAE model and were significantly diminished by Que treatment (Fig. 6A,B). Similarly, more activated GFAP^+^ astrocytes were found in the spinal cord of EAE mice as compared to normal controls as well as Que treated EAE mice, indicating that the activation of microglia and astroctyes in EAE had been attenuated by Que treatment (Fig. 6C).

## Discussion

In the present study, C57BL/6 mice immunized with MOG_35–55_ peptide display the disease symptoms associated with demyelination, glial activation, and CD4^+^ T cell infiltration in the spinal cord. These findings indicate that our EAE models are similar with previous reports and mimic the onset of MS, the T cell mediated autoimmune demyelinating disease [18]. Our study demonstrates the beneficial effects of the administration of Que, a current antipsychotic drug, on the MOG-induced EAE model. Specifically, Que treatment delays the onset, prevents deterioration of EAE symptoms, prevents demyelination, and inhibits generation of effector T cells in peripheral lymphatic organs that may results in reducing local inflammation in the spinal cord.

It has been previously reported that Que is protective against myelin breakdown in the cupriozne-induced demyelination model by up-regulation of SOD activity [16]. Our present observations provide further evidence to demonstrate the beneficial effects of Que on myelin forming OLs in EAE spinal cord (Fig 2, 5). Enhanced MBP expression and the number of CC1^+^ mature OLs found in the Que treated mice suggest that Que may protect OLs from undergoing cell death, consistent with the previous findings, which report that Que may alleviate oxidative stress, neutralize free radicals or modulate the expression and localization of the pro- and anti-apoptotic genes Bax and Bcl-X(l/s) [12], [15]. Nevertheless, we did not examine cell apoptosis in this case and cannot provide direct evidence to confirm this proposed mechanism in the current study. In trying to address the effect of Que on OL lineage, our present results demonstrate that the proliferation of NG2 positive OPCs were present in EAE mice with/without Que treatment (Figure S2), one of the prominent responses to demyelination [19]. Thus Que displays a neuroprotective capacity in EAE mice. On the other hand, Que may promote OPCs differentiate into myelin-formating OLs, especially when considering the increased number of CC1^+^ mature OLs after Que treatment (Fig. 2, 5), combined with the previous observation that Que facilitates the maturation of newly formed OLs in cultures [14]. Given that the differentiation block of OPCs as a cause for failure of remyelination in chronic MS [20], our data suggests that the therapeutic effect of Que on EAE may be due to, at least in part, the capacity to promote OPCs differentiation into mature OLs that enhances remyelination in EAE model, similar to certain growth factors such as insulin-like growth factor 1 (IGF-1) or T3 [21], [22] which have been shown to promote remyelination efficiency in EAE even the precise molecular mechanisms remain unclear.

Regarding the pathophysiology of MS and/or EAE, it has been shown that active CD4^+^ T cells may trigger local neuroinflammation and further induce demyelination [3]. In our study, we provide morphological evidences demonstrating that CD4^+^ T cells are harmful for OLs lineage as neither OLs nor MBP expression can be detected around the lesion area with the accumulation CD4^+^ T cells (Fig. 3–5). Que treatment, however, reduces the infiltration of CD4^+^ T as well as the activation of microglial and astroglial in the spinal cord of EAE mice (Fig. 6). Although the inhibitory effect of Que on microglial activation has been described in the cuprizone induced demyelination mouse model [16], our current data provide further evidence to suggest that Que has the potential capacity to reduce microglial and astroglial activation, and decrease myelin-specific T cells infiltration.

In respect with the decrease of T cell infiltration, does it due to an inhibited local immune response in CNS or alternatively a decreased effector T cell generation in peripheral immune organs? As in MS and EAE, the generation of myelin protein-specific T cells has been considered as a hallmark that triggering local inflammation and demyelination. A number of drugs, such as glatiramer acetate (GA) or lithium have been found to decrease the generation of myelin protein-specific T cells that contribute to their therapeutic effect on MS or EAE [10], [23]. In our study, we demonstrate that both CD4^+^ and CD8^+^ cell populations decreased in peripheral immune organs after Que treatment (Fig. 4B). Moreover, we found that Que inhibited either MOG_35–55_-specific immunoresponse or CD3 Ab induced non-specific T cell proliferation and the transcriptional level of IL-2 (Fig. 4C–F), a crucial cytokine to initiate and maintain T cell proliferation [24], [25]. Therefore, the decreased infiltration of CD4^+^/CD8^+^ T cells found in spinal cord after Que treatment seems mainly due to the immnunomodulatory role of Que that decreases effector T cell generation and inhibits local immunoresponsiveness. This data reveals for the first time, that Que displays an efficacy of modulating immunoreaction and may potentially be used in the treatment of other autoimmune disease beyond MS.

As an atypical antipsychotic drug, what molecular mechanisms probably underlie the immunomodulatory effects? Recently, several neurotransmitters have been considered as potent immune-modulators. For instance, serotoninergic receptors are expressed by a broad range of inflammatory cell types, including dendritic cells, helper T cells, cytotoxin T cells and so on [26], [27]. 5-HT can induce Ag-specific Th1 and cytotoxin T cell proliferation via 5-HT receptor 2 and blockade of 5-HT receptor 1 attenuates deterioration of EAE model [26], [27]. Similarly, histamine increases both ConA-dependent non-specific and TCR-mediated Ag-specific T cell proliferation via histamine receptor 2 and 1 respectively [28], [29]. Giving that Que acts as an antagonist of multiple neurotransmitter receptors, including serotonin 5-HT(1A), 5-HT(2A), dopamine D(1), D(2), histamine H(1), adrenergic alpha(1) and alpha(2) receptors [30], [31], it is possible that the antagonistic effect of Que on 5-HT receptors or other a broad range receptors may contribute to its immune-modulatory effect, especially the inhibition of effector T cell proliferation. To substantiate this speculation, extensive immunological experiments are required in further studies.

The finding that the beneficial efficacy of Que on EAE mice is particularly relevant to clinical studies, in that some patients with MS also display a variety of psychiatric symptoms, including depression or cognitive dysfunction [32], and some antipsychotic drugs, or antidepressants have been used to treat these symptoms. For example, fluoxetine, an antidepressant, has been effective in reducing lesions in relapsing MS patients [33]. Morever, oligodendrocyte dysfunction or demyelination has recently been implicated in the pathophysiology of schizophrenia, bipolar disorder and major depression [12], [34], [35]. Interestingly, all three disorders display major overlapping domains in their transcription profiles, especially genes involved in energy metabolism, inflammation and myelination [36]. Therefore, our present observations may support the notion that some psychiatric disorders such as schizophrenia, bipolar disorder or major depression may share similarities in disease mechanism with MS.

In conclusion, this study demonstrates that the atypical antipsychotic drug Que exerts immunomodulatory role and prevent mice from deterioration of EAE symptoms and demyelination. The novel effect of Que described here may lead to more effective strategies for treating not only schizophrenia, but other autoimmune diseases such as MS, and also provides new insight into pathogenesis of schizophrenia and related psychiatric disorders.

## Supporting Information

Figure S1 **Pre-treatment of Que delays the onset of EAE and relieves the symptoms.** Que pre-treatment (N = 10) was initiated 7 days before immunization, delays the onset of EAE and relieves the symptoms as compared with vehicle (N = 10) (*p*<0.05).
(TIF)

Figure S2 **Que decreases the proliferation of OPCs in spinal cord.** Ki67 (red) and NG2 (green) double immunostaining identifies the proliferating OPCs in the spinal cord. A: Ki67/NG2 double-labeled cells (arrows) are often observed in the EAE model without Que treatment, and such cells are also present in the EAE model with Que treatment (A’), however, cell numbers are diminished as compared to EAE models without Que treatment, displayed in the magnified panels (B–E, B’–E’). Scale bar, A–F = 0.2 mm.
(TIF)

Table S1 **The antibody information.**
(DOC)
