# Supplementary figures and images for: Quetiapine, an Atypical Antipsychotic, Is Protective against Autoimmune-Mediated Demyelination by Inhibiting Effector T Cell Proliferation
Source: PLoS One. 2012 Aug 13;7(8):e42746. doi: 10.1371/journal.pone.0042746 (PMC3418290; doi:10.1371/journal.pone.0042746)

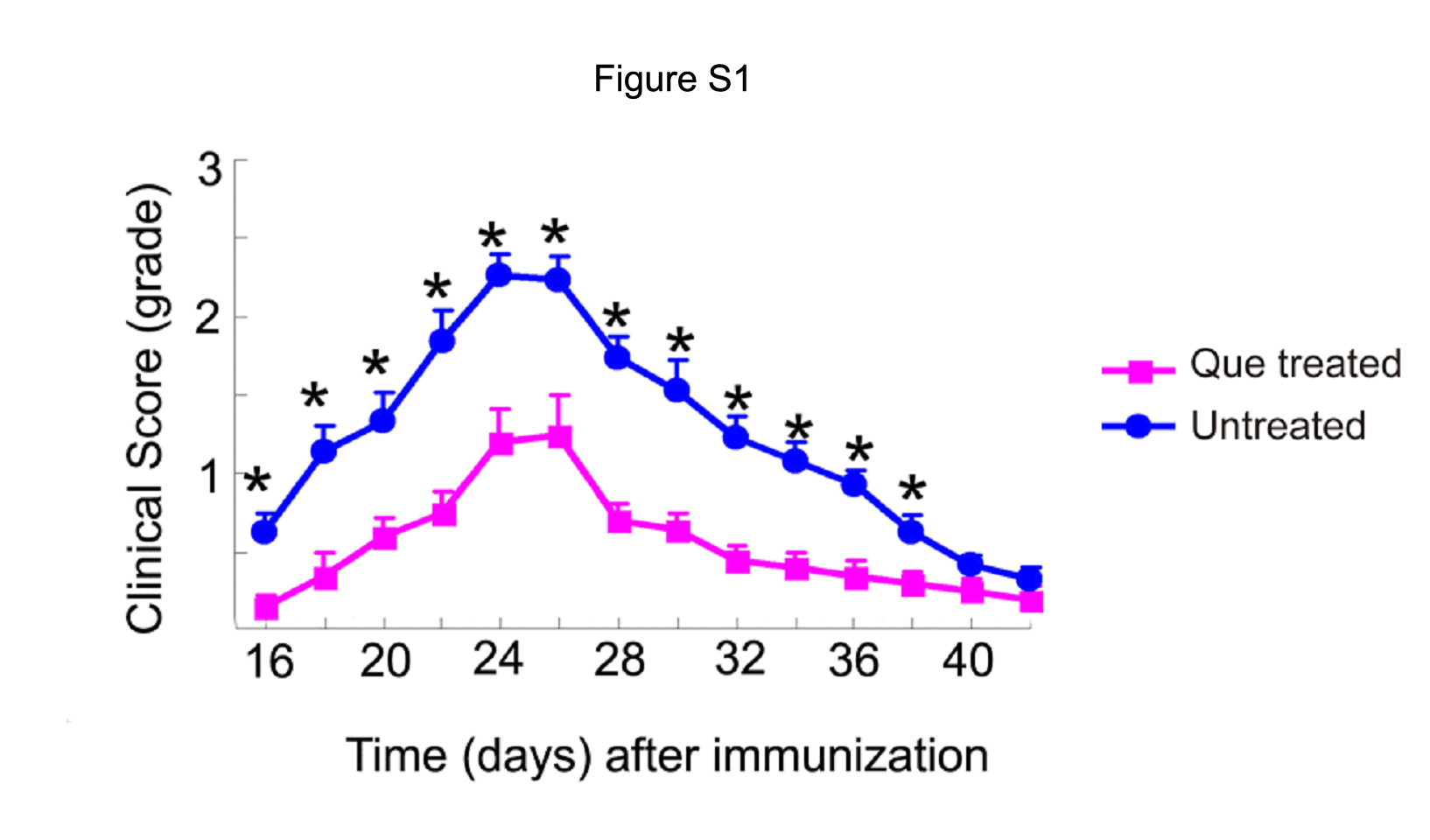

Supplement: Figure S1 — Pre-treatment of Que delays the onset of EAE and relieves the symptoms. Que pre-treatment (N = 10) was initiated 7 days before immunization, delays the onset of EAE and relieves the symptoms as compared with vehicle (N = 10) (p<0.05). (TIF) [file pone.0042746.s001.tif]

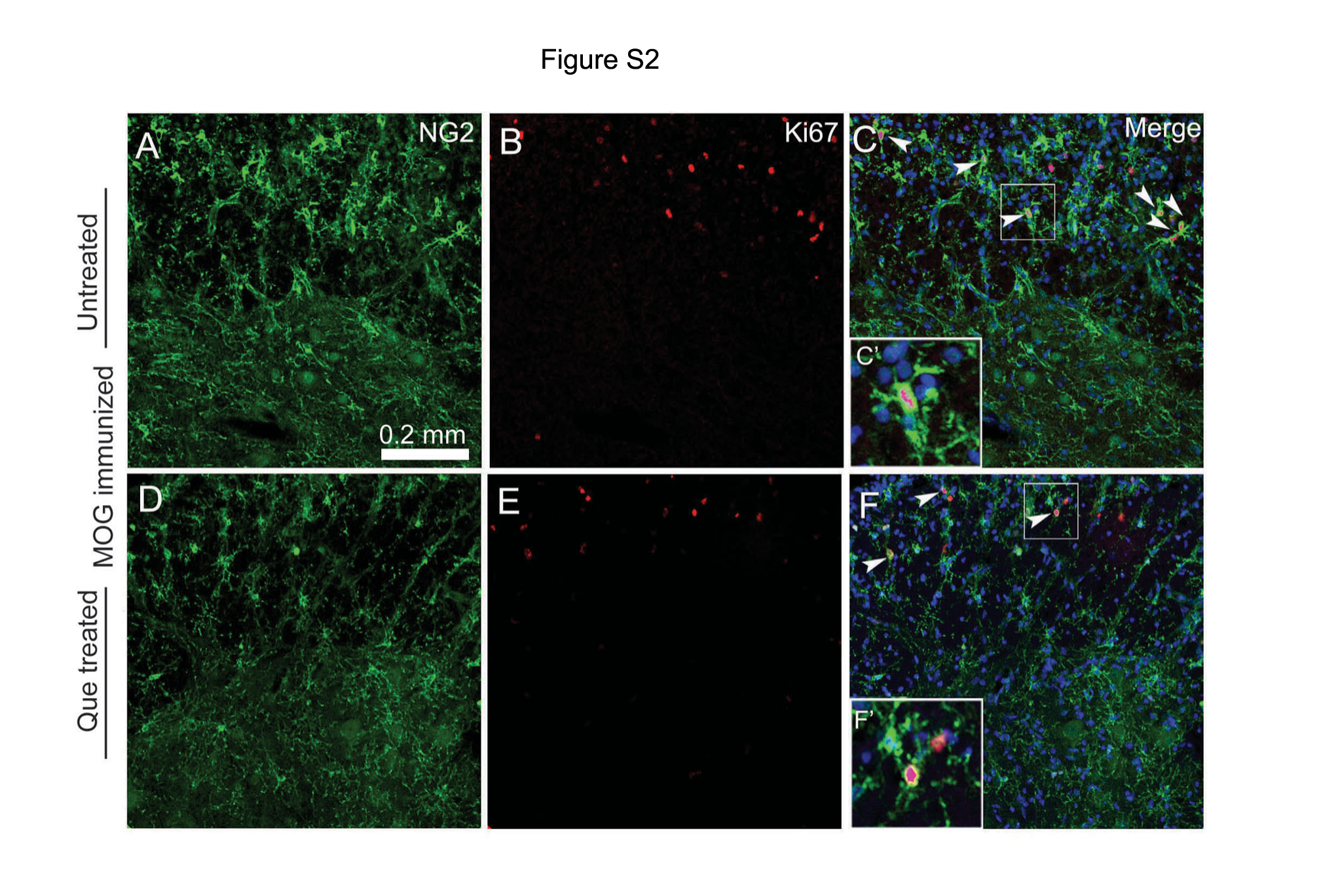

Supplement: Figure S2 — Que decreases the proliferation of OPCs in spinal cord. Ki67 (red) and NG2 (green) double immunostaining identifies the proliferating OPCs in the spinal cord. A: Ki67/NG2 double-labeled cells (arrows) are often observed in the EAE model without Que treatment, and such cells are also present in the EAE model with Que treatment (A’), however, cell numbers are diminished as compared to EAE models without Que treatment, displayed in the magnified panels (B–E, B’–E’). Scale bar, A–F = 0.2 mm. (TIF) [file pone.0042746.s002.tif]
