# Supplementary material for: Quetiapine, an Atypical Antipsychotic, Is Protective against Autoimmune-Mediated Demyelination by Inhibiting Effector T Cell Proliferation
Source: PLoS One. 2012 Aug 13;7(8):e42746. doi: 10.1371/journal.pone.0042746 (PMC3418290; doi:10.1371/journal.pone.0042746)
Supplement: Table S1 — The antibody information. (DOC) [file pone.0042746.s003.doc]

**Table S1** The antibodies

| Antigen | Clone | Supplier | Dilution | Isotype | Conjugated |
| --- | --- | --- | --- | --- | --- |
| MBP | Polyclone | Santa Cruz | 1:500 | Goat gG | Purified |
| CD4 | GK1.5 | eBioscience | 1:400 | Rat IgG | Purified |
| CD8 | eBioH35-17.2 | eBioscience | 1:400 | Rat lgG | Purified |
| CD11b | M1/70 | eBioscience | 1:400 | Rat gG | Purified |
| F4/80 | BM8 | eBioscience | 1:600 | Rat lgG | Purified |
| APC | CC1 | Calbiochem | 1:300 | Mouse IgG | Purified |
| NG2 | Polyclone | Millipore | 1:500 | Rabbit gG | Purified |
| GFAP | Polyclone | Boster | 1:500 | Rabbit IgG | Purified |
| Ki67 | SP6 | Neomarker | 1:300 | Rabbit IgG | Purified |
| Anti-Rat | Polyclone | Invitrogen | 1:800 | Donkey IgG | Alex568 |
| Anti-Rabbit | Polyclone | Invitrogen | 1:800 | Donkey IgG | Alex568 |
| Anti-Rabbit | Polyclone | Invitrogen | 1 :800 | Donkey IgG | Alex488 |
| Anti-Goat | Polyclone | Invitrogen | 1:800 | Donkey IgG | Alex568 |
| Anti-Goat | Polyclone | Zymed | 1:800 | Donkey IgG | Cy5 |
| Anit-Mouse | Polyclone | Invitrogen | 1:800 | Donkey IgG | Alex568 |
